# Glucuronolactone Alleviates Metabolic Stress Induced by High-Fat Diet in Turbot (*Scophthalmus maximus* L.)

**DOI:** 10.3390/antiox15040472

**Published:** 2026-04-10

**Authors:** Ping Wang, Luyao Zheng, Liping Zhu, Kecai Chen, Dongsheng He, Jingjing Zhao, Houguo Xu, Kangsen Mai, Yanjiao Zhang

**Affiliations:** 1 Key Laboratory of Aquaculture Nutrition and Feed, Ministry of Agriculture and Rural Affairs, The Key Laboratory of Mariculture, Ministry of Education, Ocean University of China, Qingdao 266003, China; 2 Shandong Engineering Research Center for Natural Product Metabolic Engineering and Synthetic Biology, Weifang 255178, China; 3 Yellow Sea Fisheries Research Institute, Chinese Academy of Fishery Sciences, 106 Nanjing Road, Qingdao 266071, China

**Keywords:** glucuronolactone, hepatic health, lipid metabolism, intestinal barrier, gut microbiota

## Abstract

This study aimed to investigate the ameliorative effects of glucuronolactone (GL) as a dietary additive on high-fat diet (HFD)-induced growth suppression and metabolic disorders in turbot. A 10-week feeding trial was conducted using juvenile turbot (16.7 ± 0.03 g). Two diets with different protein (%)/lipid (%) levels were formulated: PC (54/12) and NC (47/17). Based on the NC diet, three experimental diets were prepared by supplementing 200 (G200), 400 (G400), and 600 (G600) mg/kg of GL. The present results show that compared to the PC group, HFDs significantly inhibited the growth performance of turbot and induced severe metabolic disorders, hepatointestinal damage, and gut microbiota dysbiosis. Dietary GL supplementation effectively reversed these adverse effects. Specifically, compared to the NC group, GL supplementation significantly restored growth performance, enhanced non-specific immunity, and systematically improved metabolic health. In the liver, GL notably ameliorated tissue damage and downregulated key lipogenic genes (*SREBP1*, *ACC*, *FAS*, *PPARγ*), while upregulating genes involved in lipid oxidation and catabolism (*PPARα1*, *CPT1*, *ACOX1*, *HSL*, *LPL*) and lipid transport (*ApoB100*, *MTP*), thereby alleviating hepatic lipid deposition. Furthermore, GL activated the Nrf2/Keap1 antioxidant pathway, up-regulating the expression of genes such as *SOD*, *CAT*, *GPX*, and *HO-1*. It also suppressed the NF-κB-mediated inflammatory response (downregulation of *IL-1β*, *IFN-γ* and *TNF-α2*; upregulation of *IL-10* and *TGF-β2*) and the mitochondrial apoptosis pathway (increased Bcl-2/Bax ratio; downregulation of *Caspase3/7/9*), collectively mitigating oxidative damage and cellular apoptosis. Moreover, GL restored intestinal morphology, enhanced the expression of tight junction proteins (*Claudin-3*, *Claudin-7*, *ZO-1*, *Occludin*) and *MUC2*, and inhibited MLCK signaling. These improvements led to a reduction in serum D-LA levels, indicating strengthened intestinal barrier function. Concurrently, GL reshaped the gut microbiota composition by enriching beneficial bacteria such as *Akkermansia* and suppressing potential pathogens like *Listeria*. In summary, GL effectively alleviated HFD-induced growth suppression and metabolic damage in turbot by improving lipid metabolism and alleviating hepatic injury, while concurrently restoring intestinal barrier integrity and microbiota homeostasis.

## 1. Introduction

In the context of the continuously expanding global aquaculture industry, increasing dietary lipid levels has become a widely adopted nutritional strategy to reduce dependence on protein through the protein-sparing effect [1]. However, long-term feeding with high-fat diet (HFD) induces metabolic disorders in fish, leading to a series of adverse effects such as abnormal hepatic lipid deposition, oxidative stress, inflammatory responses, and microbial dysbiosis, which ultimately suppress growth performance and compromise organism health [2,3,4]. For instance, studies on rice field eel [5] and spotted sea bass [2] have demonstrated that an HFD disrupts hepatic lipid metabolism, causing lipid accumulation in the liver while inhibiting the activity of antioxidant enzymes and triggering oxidative stress. Research on hybrid groupers [6] and turbot [7] has shown that an HFD damages intestinal structure, reduces the expression of tight junction proteins such as *Claudin-3a* and *ZO-1*, thereby impairing intestinal barrier function and exacerbating inflammatory responses. Furthermore, a growing number of studies indicate that HFD may also worsen these adverse effects by altering the composition of the intestinal microbiota [8,9]. Currently, the primary approaches to ameliorate HFD-induced lipid metabolic disorders and maintain hepatointestinal health in fish involve the use of functional feed additives, such as prebiotics [8], probiotics [10,11], plant extracts [6,12], and specific nutrients [13,14].

Among the multi-tissue injuries induced by an HFD, hepatotoxicity constitutes its core pathological foundation. As the central organ for lipid metabolism, high-fat loading disrupts hepatic lipid metabolic homeostasis. This is characterized by aberrant activation of lipogenic pathways such as those mediated by SREBP1, and inhibition of catabolic pathways like PPARα-mediated fatty acid β-oxidation, ultimately leading to abnormal lipid accumulation in the liver [5]. This metabolic imbalance triggers excessive production of reactive oxygen species (ROS), causing compensatory dysfunction of the antioxidant system involving enzymes such as SOD and GPX [15]. This promotes the accumulation of lipid peroxidation products like MDA, which damage hepatocyte membrane structure and function [16]. Persistent oxidative stress further interferes with protein folding, activates the unfolded protein response (UPR), compromises endoplasmic reticulum function, and facilitates cell apoptosis through mediators like CHOP [3,17]. Concurrently, ROS and accumulated lipids can activate the NF-κB pathway, upregulating the expression of pro-inflammatory factors such as *TNF-α* and *IL-6*, thereby inducing hepatic inflammation [18]. This cascade of reactions not only directly impairs liver function but may also exacerbate intestinal barrier damage and microbiota dysbiosis via the circulatory system, leading to systemic injury [19]. Consequently, targeting the enhancement of hepatic detoxification and antioxidant capacity represents a crucial step in mitigating the toxic effects of HFDs.

Glucuronolactone (GL) is a safe, water-soluble precursor of endogenous substances, which is hydrolyzed into glucuronic acid in the liver to exert its functions [20]. Glucuronic acid serves as a critical conjugating agent in hepatic Phase II detoxification, binding to endogenous waste products (such as bilirubin), exogenous toxins, and lipid peroxidation end products to form water-soluble complexes for excretion [21]. By reinforcing this pathway, GL can indirectly alleviate hepatic metabolic burden, oxidative stress, and inflammatory responses. Furthermore, studies in mammals indicate that GL may also exert antioxidant and anti-inflammatory effects by regulating the Nrf2 and NF-κB signaling pathways [21]. Currently, GL is primarily used clinically as a clinical drug for treating liver diseases such as hepatitis and cirrhosis, while research on its application as a feed additive is limited. In livestock and poultry, supplementation with 140 mg/kg GL has been shown to increase hepatic CAT and T-AOC in laying hens, improving egg quality and alleviating liver inflammation [22]. In piglets, 200 mg/kg GL mitigated intestinal barrier damage, inflammation, and microbial dysbiosis induced by the combined challenge of porcine reproductive and respiratory syndrome virus (PRRSV) and deoxynivalenol (DON) [23]. In aquatic species, an early study demonstrated that 200 mg/kg GL improved lipid metabolism, immune function, and antioxidant capacity in Nile tilapia [24]. Additionally, it reported that 200–400 mg/kg GL enhanced hepatointestinal health and resistance to *Aeromonas hydrophila* infection in *Pelodiscus sinensis* [25]. Despite the limited number of studies, GL exhibits potential as a functional feed additive in aquaculture.

Turbot (*Scophthalmus maximus* L.) is an economically important marine fish species in northern coastal China. This study investigated the effects of graded GL supplementation in an HFD on turbot. We systematically evaluated its potential to alleviate HFD-induced growth suppression, lipid metabolism disorders, liver injury, intestinal barrier impairment, and gut microbiota dysbiosis. The findings aim to provide a theoretical and empirical basis for the application of GL as a novel functional feed additive in aquaculture.

## 2. Materials and Methods

### 2.1. Diets and Husbandry Protocol

By adjusting the inclusion levels of fishmeal and soybean oil, five isoenergetic diets were formulated, including a normal-fat diet (PC, 54% crude protein and 12% crude fat) and a high-fat diet (NC, 47% crude protein and 17% crude fat) supplemented with 5% soybean oil. Based on the NC diet, three graded levels of Glucuronolactone (Purity > 95%, Zhucheng Haotian Pharmaceutical Co., Ltd. (Weifang, China)) were supplemented at 200, 400, and 600 mg/kg of diet, designated as G200, G400, and G600, respectively (Table 1). All dry ingredients were ground, passed through a 60-mesh sieve, and mixed stepwise according to the formulation. Fish oil and 23% distilled water were then added, and the mixture was homogenized thoroughly using a feed mixer. The moist mash was pelleted into 3 mm diameter pellets using a pelletizer. After drying at 55 °C in a forced-air oven for 8 h, the diets were packed and stored at −20 °C until use.

Juvenile turbot from the same cohort were acclimated for 14 days before the experiment. A total of 600 fish (16.7 ± 0.03 g) were randomly stocked into fifteen 200 L tanks (40 fish per tank) in a flow-through seawater system for a 10-week feeding trial. Each diet was assigned to three replicate tanks in a completely random design. Fish were fed to apparent satiation twice daily (07:00 and 19:00). Prior to each feeding, about two-thirds of the water was renewed and feces were siphoned. Uneaten feed was removed promptly after feeding. Water quality was monitored daily. Temperature was measured with a thermometer, pH with a pH meter (pH-100, LICHEN, Xiamen, China), salinity with a handheld refractometer (WZ-211, ATAGO, Tokyo, Japan), and dissolved oxygen with a dissolved oxygen meter (JPB-607A, LEICI, Shanghai, China). The conditions were maintained at 13–21 °C, pH 7.5–7.8, salinity 26–32 ppt, and dissolved oxygen >7.3 mg/L.

### 2.2. Sample Collection

At the end of the trial, fish were fasted for 24 h and anaesthetised with eugenol (1:10,000, *v*/*v*). Total biomass and individual counts per tank were recorded for growth evaluation. Two fish per tank were randomly selected for morphometric measurement. For tissue sampling, three fish per tank were dissected to collect posterior intestine and liver. These tissues were fixed in Bouin’s solution for 24 h and then transferred to 75% ethanol for subsequent histological analysis (HE staining). Another three fish per tank were used for gene expression analysis, posterior intestine and liver were collected, immediately placed in sterile RNase-free 2 mL cryotubes, flash-frozen in liquid nitrogen, and stored at −80 °C. For gut microbiota profiling, the posterior intestine from three additional fish per tank was aseptically sampled and preserved using the same freezing protocol. Blood was drawn from the caudal vein of all sampled fish. After clotting at 4 °C for 4 h, samples were centrifuged (4000× *g*, 10 min, 4 °C) to obtain serum, which preserved using the same freezing protocol for biochemical assays. Dorsal muscle from all sampled individuals per tank was pooled and kept at −20 °C for proximate composition analysis. All sampling procedures were performed under aseptic conditions.

### 2.3. Chemical Analysis

Proximate composition was determined following the AOAC standard methods. Crude protein content was quantified using the Kjeldahl method (Kjeltec 8400, FOSS, Höganäs, Sweden), crude lipid was extracted with the Soxhlet method (Soxtec 8000, FOSS, Sweden), moisture was measured by drying samples to constant weight at 105 °C for 24 h, and ash content was determined by incineration at 550 °C in a muffle furnace for 8 h until constant weight was achieved.

### 2.4. Serum Parameters and Hepatic Antioxidant Parameters

Serum Lysozyme (LZM) and D-lactic acid (D-LA) concentrations were determined using ELISA kits purchased from Shanghai Fankew Industrial Co., Ltd. (Shanghai, China). Serum glucose (GLU, A154-1-1), total cholesterol (TC, A111-1-1), acid phosphatase (ACP, A060-2-2), triglycerides (TG, A110-1-1), alkaline phosphatase (ALP, A059-2-2), aspartate aminotransferase (AST, C010-2-1) and alanine aminotransferase (ALT, C009-2-1) were assayed with kits from Nanjing Jiancheng Bioengineering Institute (Nanjing, China). Additionally, hepatic malondialdehyde (MDA, A003-1-2) and total antioxidant capacity (T-AOC, A015-2-1) were determined using kits from the same manufacturer.

### 2.5. Morphological Analysis

Following a previously described protocol [26], tissues were dehydrated through a graded ethanol series, embedded, and sectioned at 5 μm (Leica RM2235, Nussloch, Germany). After hematoxylin-eosin (HE) staining, sections were observed and photographed under an optical microscope (DP 72, Olympus, Tokyo, Japan) equipped with a digital imaging system (CellSens Standard 2.1 software). Villus perimeter ratio was quantified using Image-Pro Plus 6.0 software.

### 2.6. RNA Extraction and Real-Time Quantitative PCR Analysis

All reagents used for molecular analyses were sourced from Accurate Biotechnology Co., Ltd. (Changsha, China). Total RNA was extracted from tissues using the AG RNAex Pro Reagent (AG21102). RNA integrity and purity were assessed by 1.2% denaturing agarose gel electrophoresis and a NanoDrop 2000 spectrophotometer (Thermo Fisher Scientific, Waltham, MA, USA), respectively. cDNA was synthesized using the Evo M-MLV Reverse Transcription Premix Kit (AG11728) containing gDNA remover. Quantitative real-time PCR was performed with the SYBR Green Pro Taq HS qPCR Kit (AG11701) on a CFX96 Touch Real-Time PCR Detection System (Bio-Rad, Hercules, CA, USA) following the manufacturer’s instructions. Primers were designed and synthesized by Sangon Biotech (Shanghai, China), and their sequences are provided in Supplementary Table S1. Gene expression levels were normalized to β-actin and calculated using the 2^−ΔΔCt^ method [27].

### 2.7. Intestinal Microbiota DNA Extraction and Sequencing

Intestinal microbiota samples were aseptically collected by scraping the hindgut mucosa in a laminar flow hood. All samples were flash-frozen in liquid nitrogen and then sent to Novogene Bioinformatics Technology Co., Ltd. (Beijing, China) for DNA extraction, library construction, and sequencing. The specific procedures were as follows: Genomic DNA was extracted from the mucosal samples, and the V4 hypervariable region of the 16S rRNA gene was amplified using the primers 515F/806R. The resulting amplicons were subjected to paired-end sequencing on the Illumina NovaSeq platform. The raw sequencing data were processed using the following bioinformatics pipeline: Sequences were demultiplexed based on sample-specific barcodes, and paired-end reads were merged using FLASH (v1.2.7). Quality filtering was performed via the QIIME (v1.9.1) pipeline to obtain high-quality clean tags [28]. Chimeric sequences were then identified and removed using the UCHIME algorithm [29]. Denoising and generation of amplicon sequence variants (ASVs) were conducted with the DADA2 (version 1.16.0) package [30]. Taxonomic assignment of representative ASV sequences was carried out against the SILVA database using the RDP classifier (v2.2) [31], followed by removal of sequences originating from mitochondria, chloroplasts, and streptophytes. Based on the processed data, microbial community structure was evaluated by α-and β-diversity analyses, and the MetagenomeSeq (v1.38.0) tool was applied to identify taxa with significantly differential abundance among the experimental groups.

### 2.8. Calculation and Statistical Analysis

For calculation and statistical analysis, the following equations and definitions are used:

Weight gain rate (WGR, %) = 100 × (*W_f_* − *W_i_*)/*W_i_*

Specific growth rate (SGR, %/day) = 100× (Ln *W_f_* − Ln *W_i_*)/t

Daily Feed intake (FI, %/day) = 100 × *I_d_*/[(*W_f_* + *W_i_*)/2]/t

Feed conversion rate (FCR) = *I_d_*/(*W_f_* − *W_i_*)

Survival rate (SR, %) = 100 × *N_t_*/*N*_0_

Condition factor (CF, 100 g/cm^3^) = 100 × *W_f_*/*body length*^3^

Hepatosomatic index (HSI, %) = 100 × *liver weight*/*W_f_*

*W_f_*, final body weight; *W_i_*, initial body weight; t, experimental duration; *I_d_*, feed intake; *N_t_*, final number of fish; *N_0_*, initial number of fish.

All data were analyzed using SPSS (version 26.0, IBM, Armonk, NY, USA) and expressed as mean ± SE. After verifying normality (Shapiro–Wilk test) and the homogeneity of variance (Levene’s test), one-way ANOVA followed by Tukey’s post hoc test was applied to compare differences among dietary groups. Statistical significance was set at *p* < 0.05.

## 3. Results

### 3.1. Growth Performance

Compared with the PC group, the NC group showed significant decreases in FBW, WGR, SGR, FI, and CF (*p* < 0.05). Supplementation with GL improved all these parameters. Specifically, CF increased significantly in all GL-supplemented groups (*p* < 0.05), while WGR and SGR in the G600 group and FI in the G400 group were significantly higher than those in the NC group and recovered to levels comparable with the PC group (*p* < 0.05). No significant differences were observed among groups in SR, FCR, or HSI (*p* > 0.05). However, a declining trend in HSI was noted in the GL-supplemented groups (Table 2).

### 3.2. Proximate Composition of Muscle

The high-fat diet significantly decreased muscle crude protein content (*p* < 0.05) but did not affect moisture, lipid, or ash levels. Although GL supplementation caused no significant changes, a dose-dependent trend was noted: with increasing GL levels, moisture and crude protein content tended to increase, whereas lipid content showed a tendency to decrease (Table 3).

### 3.3. Serum Biochemical Parameters

The high-fat diet (NC) significantly increased serum levels of ALP, TC, TG, GLU, ALT, and D-LA, while decreasing LZM activity compared to the PC group (*p* < 0.05). GL supplementation effectively reversed these alterations. Specifically, all GL-supplemented groups showed significant changes in LZM, ALP, TC, TG, GLU, and AST (*p* < 0.05). Additionally, ALT and D-LA levels in the G400 and G600 groups were significantly lower than those in the NC group (*p* < 0.05). Moreover, compared to the NC group, G400 further significantly increased ACP activity (*p* < 0.05). The changes in LZM, ALP, TC, TG, GLU, ALT, and D-LA exhibited a clear dose-dependent relationship with GL supplementation, with significant differences observed in LZM, TC, and TG levels between G600 and G200, as well as in TG between G400 and G200 (*p* < 0.05) (Table 4).

### 3.4. Liver Morphology

**Figure 1:**
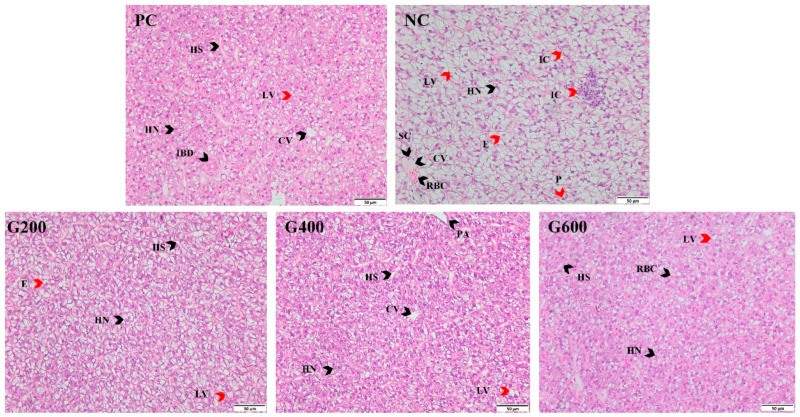
Effects of glucuronolactone on liver morphology (×400 magnification). Black arrows indicate normal structures: HN, hepatocyte nucleus; HS, hepatic sinusoid; CV, central vein; PA, portal area; IBD, interlobular bile duct; RBC, red blood cell; SC, squamous cell. Red arrows indicate pathological changes: LV, lipid vacuoles, characterized by clear, round vacuoles of varying sizes within the cytoplasm; E, cellular edema, manifested as swollen hepatocytes with pale cytoplasm; P, nuclear pyknosis, displaying condensed, shrunken, and deeply stained nuclei; IC, inflammatory cell infiltration, presented as aggregation of small, deeply stained cells.

HE staining results showed that the high-fat diet (NC group) induced typical fatty liver pathology in turbot, manifested by extensive vacuolation in the hepatocyte cytoplasm, cellular swelling, disorganized hepatic cord arrangement, congestion in hepatic sinusoids and central veins, nuclear pyknosis, and localized inflammatory infiltration. Compared with the NC group, the liver histopathology in glucuronolactone-supplemented groups was markedly improved. Specifically, no significant difference was observed between the G400 and G600 groups, and their tissue structure was largely restored to a level comparable to that of the PC group (Figure 1).

### 3.5. Hepatic Oxidative Stress

**Figure 2:**
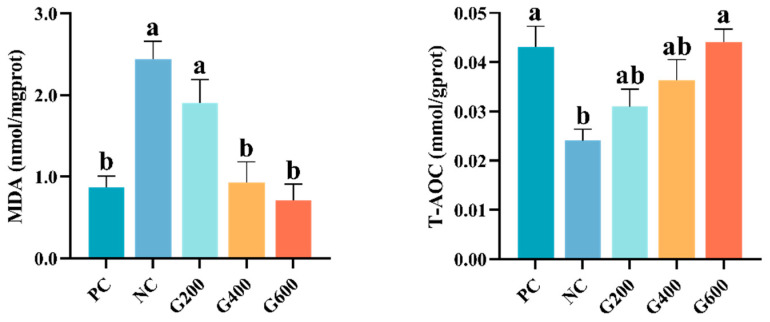
Effects of glucuronolactone on hepatic MDA content and T-AOC. MDA, Malondialdehyde; T-AOC, Total antioxidant capacity. Values are shown as the mean ± SE (*n* = 3). Different lowercase letters above the bars indicate statistically significant differences (*p* < 0.05).

As shown in Figure 2, compared to the PC group, HFDs significantly increased hepatic MDA content and decreased T-AOC (*p* < 0.05). GL supplementation significantly reversed this trend. MDA levels were significantly reduced in the G400 and G600 groups, while T-AOC was significantly elevated in the G600 group and restored to the level of the PC group (*p* < 0.05).

### 3.6. Expression of Genes Related to Hepatic Antioxidant Response, Inflammation, and Apoptosis

**Figure 3:**
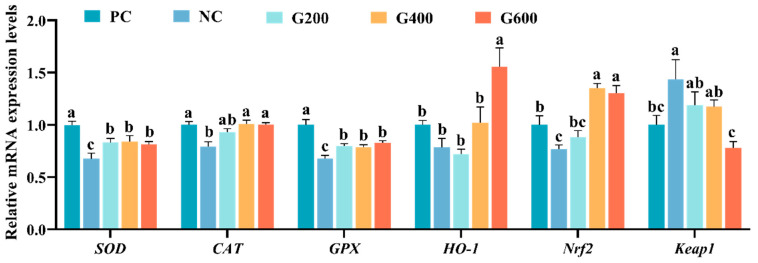
Effects of glucuronolactone on hepatic antioxidant capacity. Values are shown as the mean ± SE (*n* = 3). SOD, Superoxide Dismutase; CAT, Catalase; GPX, Glutathione Peroxidase; HO-1, Heme Oxygenase-1; Nrf2, Nuclear Factor Erythroid 2-Related Factor 2; Keap1, Kelch-like ECH-Associated Protein 1. Different lowercase letters above the bars indicate statistically significant differences (*p* < 0.05).

The effects of GL on hepatic antioxidant capacity are shown in Figure 3. Compared with the PC group, the expression levels of *SOD*, *CAT*, and *Nrf2* were significantly decreased, while *Keap1* was markedly increased in the NC group (*p* < 0.05). GL supplementation effectively reversed these alterations induced by the high-fat diet. Specifically, compared with the NC group, *SOD* and *GPX* were significantly elevated in all GL-supplemented groups (*p* < 0.05). The expression of *CAT* and *Nrf2* was significantly increased in the G400 and G600 groups (*p* < 0.05). Furthermore, the G600 group also significantly enhanced *HO-1* expression and reduced *Keap1* expression levels (*p* < 0.05).

**Figure 4:**
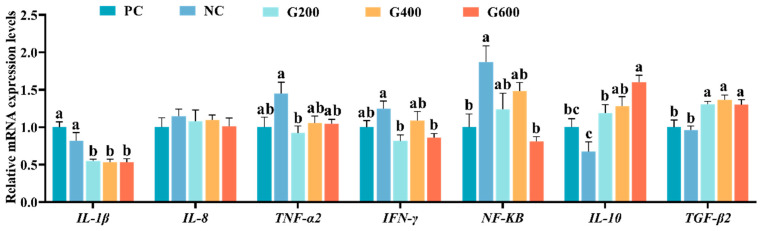
Effects of glucuronolactone on hepatic inflammatory response. Values are shown as the mean ± SE (*n* = 3). IL-1β, Interleukin-1 Beta; IL-8, Interleukin-8; TNF-α, Tumor Necrosis Factor Alpha; IFN-γ, Interferon Gamma; NF-κB, Nuclear Factor Kappa-B; IL-10, Interleukin-10; TGF-β2, Transforming Growth Factor Beta 2. Different lowercase letters above the bars indicate statistically significant differences (*p* < 0.05).

The effects of GL on hepatic inflammatory response are shown in Figure 4. Compared with the PC group, *NF-κB* expression was significantly increased in the NC group (*p* < 0.05). Although *TNF-α2* and *IFN-γ* showed an upward trend and *IL-10* a downward trend, these changes were not statistically significant (*p* > 0.05). Relative to the NC group, all GL-supplemented groups exhibited significantly reduced *IL-1β* expression and significantly elevated levels of *IL-10* and *TGF-β2* (*p* < 0.05). Following GL supplementation, expressions of *TNF-α2*, *IFN-γ*, and *NF-κB* generally decreased. Specifically, *TNF-α2* and *IFN-γ* in the G200 group, as well as *IFN-γ* and *NF-κB* in the G600 group, were significantly lower than those in the NC group (*p* < 0.05).

**Figure 5:**
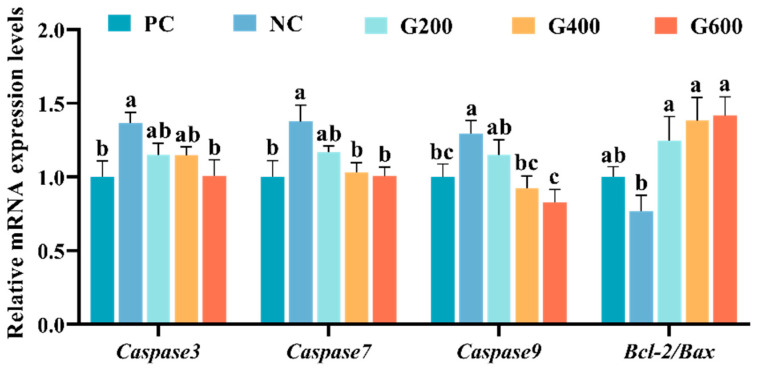
Effects of glucuronolactone on hepatic apoptosis. Values are shown as the mean ± SE (*n* = 3). Caspase, Cysteine Aspartate-Specific Protease; Bcl-2/Bax, B-Cell Lymphoma 2/Bcl-2-Associated X Protein. Different lowercase letters above the bars indicate statistically significant differences (*p* < 0.05).

The effects of GL on hepatic apoptosis are shown in Figure 5. Compared with the PC group, the high-fat diet (NC) significantly increased the expression of *Caspase3*, *Caspase7*, and *Caspase9* (*p* < 0.05), while the *Bcl-2*/*Bax* ratio showed a decreasing trend that was not statistically significant. GL supplementation dose-dependently reversed these changes. Specifically, *Caspase3* was significantly reduced in the G600 group (*p* < 0.05). *Caspase7* and *Caspase9* were significantly decreased in both the G400 and G600 groups (*p* < 0.05). Meanwhile, the *Bcl-2*/*Bax* ratio was significantly elevated in all GL-supplemented groups (*p* < 0.05).

### 3.7. Expression of Hepatic Lipid Metabolism-Related Genes

**Figure 6:**
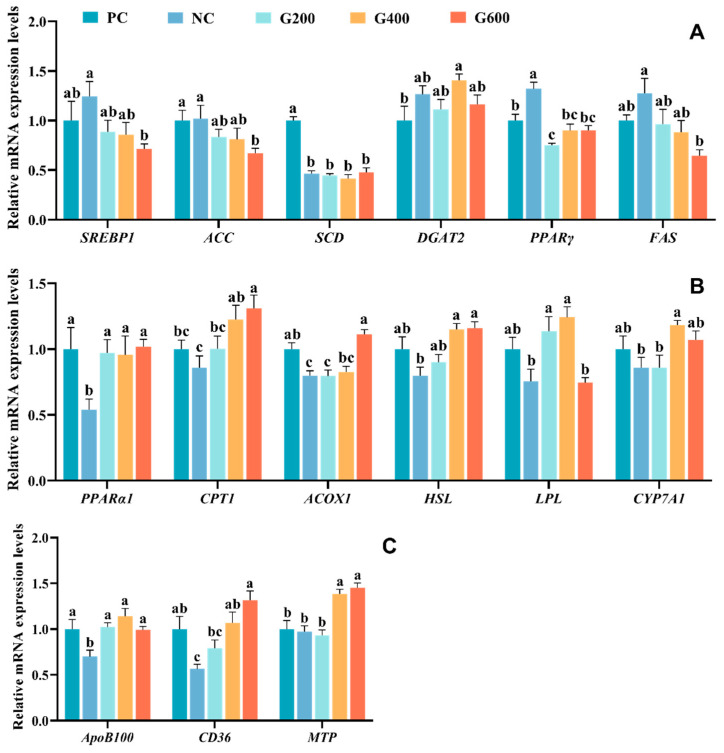
Effects of glucuronolactone on hepatic lipid metabolism: (**A**) lipid synthesis; (**B**) lipid catabolism; (**C**) lipid transport. Values are shown as the mean ± SE (*n* = 3). SREBP1, Sterol Regulatory Element-Binding Protein 1; ACC, Acetyl-CoA Carboxylase; SCD, Stearoyl-CoA Desaturase; DGAT2, Diacylglycerol O-Acyltransferase 2; PPARγ, Peroxisome Proliferator-Activated Receptor Gamma; FAS, Fatty Acid Synthase; PPARα, Peroxisome Proliferator-Activated Receptor Alpha; CPT1, Carnitine Palmitoyltransferase 1; ACOX, Acyl-CoA Oxidase; HSL, Hormone-Sensitive Lipase; LPL, Lipoprotein Lipase; CYP7A1, Cholesterol 7α-Hydroxylase; ApoB100, Apolipoprotein B100; CD36, Cluster of Differentiation 36; MTP, Microsomal Triglyceride Transfer Protein. Different lowercase letters above the bars indicate statistically significant differences (*p* < 0.05).

Regarding lipid transport (Figure 6A), the high-fat diet (NC) significantly up-regulated *PPARγ* expression and significantly down-regulated *SCD* expression (*p* < 0.05). Although *SREBP1*, *DGAT2*, and *FAS* showed an upward trend, the differences were not statistically significant (*p* > 0.05). Following GL supplementation, the expression of *ACC*, *SREBP1*, and *FAS* decreased in a dose-dependent manner with increasing GL levels, with the G600 group being significantly lower than the NC group (*p* < 0.05). *PPARγ* expression was significantly reduced in the G200 group (*p* < 0.05). GL supplementation had no significant effect on the expression of *SCD* and *DGAT2*.

Regarding lipid catabolism (Figure 6B), the high-fat diet (NC) significantly down-regulated the expression of *PPARα1* and *ACOX1* (*p* < 0.05), while *CPT1*, *HSL*, *LPL*, and *CYP7A1* showed non-significant decreasing trends (*p* > 0.05). GL supplementation effectively reversed these alterations. Specifically, *ACOX1* expression was significantly increased in the G600 group, and *CYP7A1* expression was significantly elevated in the G400 group (*p* < 0.05). *LPL* expression was up-regulated in the G200 and G400 groups, and *CPT1* and *HSL* expression were enhanced in the G400 and G600 groups (*p* < 0.05). Furthermore, *PPARα1* expression was significantly up-regulated in all GL-supplemented groups (*p* < 0.05).

Concerning lipid transport (Figure 6C), the high-fat diet (NC) significantly down-regulated the expression of *ApoB100* and *CD36* (*p* < 0.05). Following GL supplementation, *ApoB100* expression was significantly increased in all GL-supplemented groups, and *CD36* expression was significantly higher in the G400 and G600 groups compared to the NC group (*p* < 0.05). *MTP* expression showed no significant differences among the PC, NC, and G200 groups but was significantly up-regulated in the G400 and G600 groups (*p* < 0.05).

### 3.8. Intestinal Morphology

**Figure 7:**
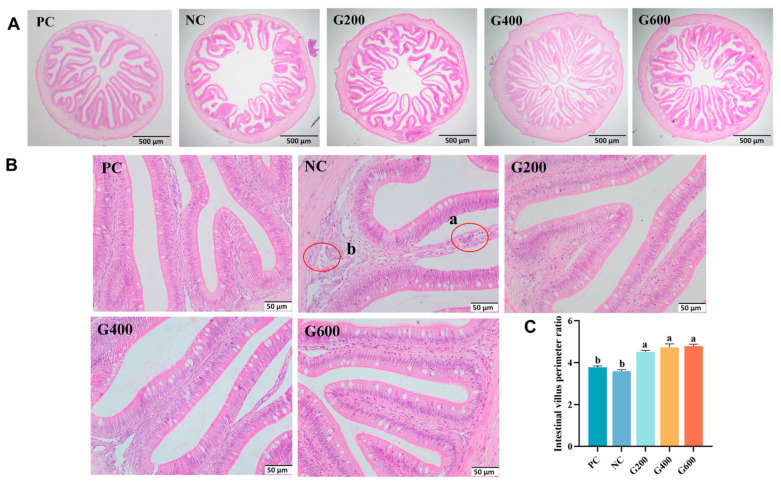
Effects of glucuronolactone on intestinal morphology: (**A**) ×40 magnification; (**B**) ×400 magnification; (**C**) intestinal villus perimeter ratio. (a) Inflammatory cell aggregation, presented as accumulation of small, deeply stained cells within the lamina propria; (b) congestion, manifested as dilated blood vessels filled with red blood cells in the submucosa. Different lowercase letters above the bars indicate statistically significant differences (*p* < 0.05).

As shown in Figure 7, compared with the PC group, the NC group exhibited disorganized intestinal villi, accompanied by fusion, atrophy, shedding, and widening of the lamina propria. At higher magnification, localized inflammatory cell infiltration and submucosal congestion were observed in the NC group. Supplementation with GL improved these morphological abnormalities and significantly increased the intestinal villus perimeter ratio (*p* < 0.05).

### 3.9. Expression of Intestinal Barrier-Related Genes

**Figure 8:**
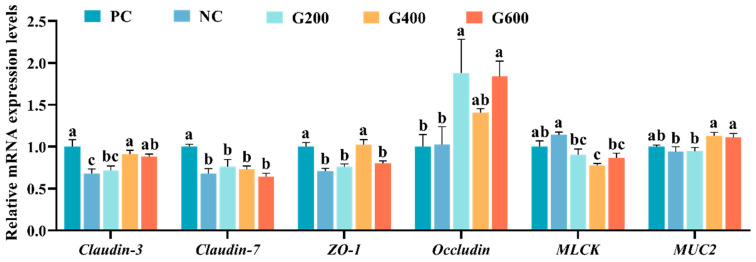
Effects of glucuronolactone on intestinal barrier function. Values are shown as the mean ± SE (*n* = 3). ZO-1, Zonula Occludens-1; MLCK, Myosin Light Chain Kinase; MUC2, Mucin 2. Different lowercase letters above the bars indicate statistically significant differences (*p* < 0.05).

Compared to the PC group, the NC group showed significantly reduced expression of *Claudin-3*, *Claudin-7*, and *ZO-1* (*p* < 0.05), while *MUC2* and *MLCK* exhibited non-significant decreasing and increasing trends (*p* > 0.05), respectively. GL supplementation increased *Claudin-3* and *MUC2* in G400 and G600, elevated *ZO-1* in G400, and raised *Occludin* in G200 and G600 relative to PC (*p* < 0.05). *MLCK* decreased in all GL-treated groups, with G400 significantly lower than NC (*p* < 0.05). *Claudin-7* remained unaffected by GL (Figure 8).

### 3.10. Gut Microbiota

**Figure 9:**
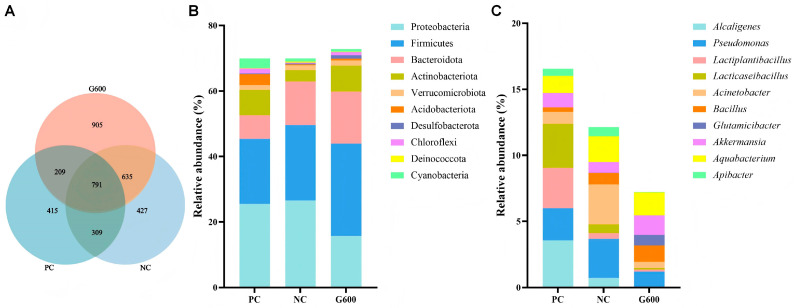
Structure and taxonomic composition of the intestinal microbiota: (**A**) ASV Venn diagram; (**B**) top 10 phyla; (**C**) top 10 genera.

**Figure 10:**
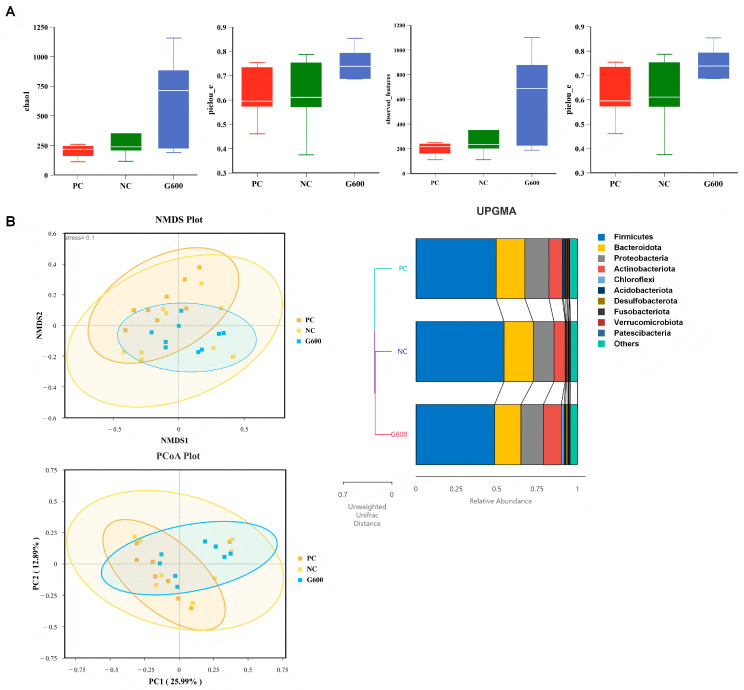
Diversity and composition of the gut microbiota: (**A**) α-diversity; (**B**) β-diversity (based on Unweighted UniFrac distance).

Rarefaction and species accumulation curves confirmed adequate sequencing depth (Figure S1). A total of 791 ASVs were shared across groups, with unique ASVs of 415 (PC), 427 (NC), and 905 (G600) (Figure 9A). At the phylum level, Proteobacteria, Firmicutes, and Bacteroidota dominated. The top 10 genera included *Alcaligenes*, *Pseudomonas*, *Lactiplantibacillus*, *Lacticaseibacillus*, *Acinetobacter*, *Bacillus*, *Glutamicibacter*, *Akkermansia*, *Aquabacterium*, and *Apibacter* (Figure 9B,C). No significant differences in α- or β-diversity were detected among groups (Figure 10) (*p* > 0.05).

**Figure 11:**
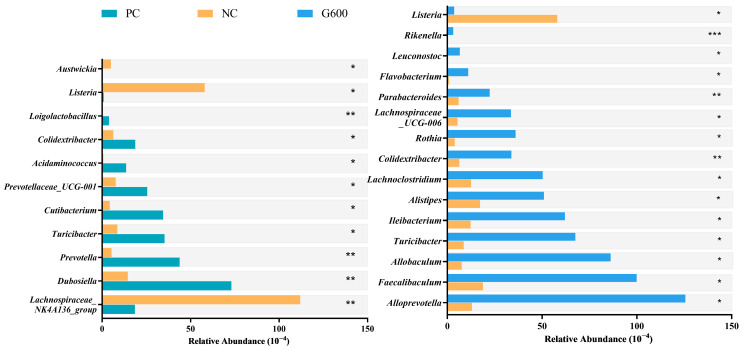
Differential abundances of gut microbiota at the genus level identified by MetagenomeSeq: * *p* < 0.05, ** *p* < 0.01, and *** *p* < 0.001.

MetagenomeSeq analysis (Figure 11) revealed that compared with the PC group, the abundances of *Lachnospiraceae_NK4A136_group*, *Listeria*, and *Austwickia* were significantly increased in the NC group, whereas the abundances of *Dubosiella*, *Prevotella*, *Turicibacter*, *Cutibacterium*, *Prevotellaceae_UCG-001*, *Acidaminococcus*, *Colidextribacter*, and *Loigolactobacillus* were significantly decreased. Compared with the NC group, the abundance of *Listeria* was significantly reduced in the GL supplemented groups. Meanwhile, the abundances of *Alloprevotella*, *Faecalibaculum*, *Allobaculum*, *Turicibacter*, *Ileibacterium*, *Alistipes*, *Lachnoclostridium*, *Colidextribacter*, *Rothia*, *Lachnospiraceae_UCG-006*, *Parabacteroides*, *Flavobacterium*, *Leuconostoc*, and *Rikenella* were significantly increased.

## 4. Discussion

In intensive aquaculture, HFDs are widely adopted to reduce feed costs and nitrogen emissions. However, excessive lipid intake often surpasses the metabolic threshold of fish, leading to suppressed growth performance and a series of health issues [3,4]. Early studies on turbot have demonstrated that high dietary lipid levels negatively affect growth performance, feed utilization, and lipid metabolism [32,33]. In this study, the NC group exhibited significantly lower FBW, WGR, and SGR compared to the PC group, consistent with findings in species such as turbot [34], spotted sea bass [35], and black sea bream [36] under high-fat stress. The primary reasons include reduced feed palatability and metabolic disorders induced by excessive dietary lipids [37]. Specifically, an HFD suppresses intestinal digestive enzyme activity, impairing nutrient absorption efficiency [37], while lipotoxicity disrupts normal hepatocyte function, ultimately compromising growth performance [5]. As a hepatoprotective and detoxifying agent, glucuronolactone (GL) has been demonstrated to promote growth in Chinese soft-shelled turtle [25] and Nile tilapia [24]. Consistent with these reports, dietary GL supplementation in this study significantly improved the WGR, SGR, and FI of turbot fed an HFD, indicating its efficacy in alleviating HFD-induced growth suppression. Similar to the mechanisms observed with hepatoprotective agents like silymarin and curcumin in aquatic species [38,39], the growth-promoting effect of GL may stem from its positive modulation of the overall metabolic state and feeding behavior. On one hand, as a hepatic detoxifying drug, GL may enhance phase II detoxification pathways, thereby mitigating metabolic liver injury caused by HFDs and improving energy allocation [21]. On the other hand, by ameliorating the metabolic environment, GL may further regulate feeding behavior or improve feed palatability, providing a favorable basis for growth recovery. Notably, although studies in piglets [23] and Chinese soft-shelled turtle [25] reported that GL simultaneously enhances digestive enzyme activity and improves feed utilization efficiency, no significant reduction in FCR was observed in the present study. This discrepancy may be attributed to the “context-dependent” nature of additive efficacy, where the primary targets and physiological effects of the same additive may vary in priority across different species or dietary backgrounds [40,41]. In summary, under the experimental conditions of this study, GL likely alleviates HFD-induced growth suppression primarily through its detoxification function to relieve hepatic metabolic stress, coupled with an increase in FI.

Changes in serum non-specific immune indicators more directly reflect the protective effects of GL. As a core enzyme in immune defense, LZM activity was significantly reduced in the NC group, consistent with previous studies showing that HFDs suppress immune function in turbot [3]. However, GL supplementation effectively reversed this trend, with LZM activity exhibiting a clear dose-dependent increase alongside rising additive levels. This result strongly indicates that GL can ameliorate HFD-induced immune suppression by enhancing the activity of this key immune enzyme. ACP and ALP are involved in material metabolism and immune responses [42] ACP, a marker enzyme of lysosomes, reflects enhanced macrophage activity and activation of the innate immune system when its activity is elevated [43]. ALP is primarily active in tissue cells, especially in the liver, and its serum level indirectly reflects cell membrane permeability [44]. In this study, the NC group exhibited abnormally elevated ALP activity alongside a non-significant increase in ACP, aligning with response patterns observed in zebrafish [15] and turbot [3]. These results suggest that the high-fat environment triggered immune stress responses and likely induced severe liver damage. GL supplementation successfully restored ALP activity to stable levels similar to those in the PC group and further enhanced ACP activity. This bidirectional regulatory pattern, downregulating an aberrant stress indicator (ALP) and upregulating a defensive activity marker (ACP), is consistent with reported mechanisms in Chinese soft-shelled turtle, where GL enhances host defense by modulating immune factors [25]. This indicates that GL effectively mitigates excessive immune stress induced by HFDs. In conclusion, appropriate doses of GL can systematically regulate LZM, ACP, and ALP activities, effectively alleviating HFD-induced immune stress and potential liver injury while enhancing the innate immune defense function of turbot.

The liver, as the central regulator of lipid metabolism, plays a critical role in maintaining metabolic homeostasis in fish. In this study, serum levels of TC, TG, and GLU were significantly elevated in the NC group of turbot. Concurrently, HE staining of liver tissue revealed numerous lipid vacuoles, consistent with the pathological features of hepatic steatosis induced by HFDs [15,45,46]. These findings indicate that an HFD disrupts lipid metabolic balance and promotes hepatic lipid accumulation in turbot. However, GL supplementation demonstrated dose-dependent ameliorative effects, significantly alleviating HFD-induced liver structural damage and dyslipidemia. This aligns with previous studies in laying hens [47] and Chinese soft-shelled turtles [25], where GL lowered blood lipid levels and improved metabolic balance. Furthermore, similar to most existing research, HFDs in this study activated the SREBP1-mediated adipogenic pathway while suppressing the PPARα-regulated β-oxidation pathway, establishing a metabolic imbalance characterized by enhanced lipid synthesis and reduced lipid catabolism [45,48]. GL intervention systematically reversed this imbalance. On one hand, GL downregulated the expression of *SREBP1*, thereby inhibiting its downstream lipogenic genes such as *ACC* and *FAS*, which are involved in de novo fatty acid synthesis [49]. On the other hand, it upregulated the expression of lipid catabolism related genes *PPARα1*, *CPT1*, and *ACOX1*, enhancing mitochondrial β-oxidation and fatty acid degradation [50]. Simultaneously, *ApoB100* and *MTP* are key components in lipoprotein assembly and lipid transport, while *CD36* facilitates fatty acid transmembrane transport [51]. GL also increased the expression of lipid transport-related genes *ApoB100*, *CD36*, and *MTP*, promoting the export of lipids from the liver and thereby reducing hepatic lipid burden. Beyond transcriptional regulation, studies suggest that GL may further promote triglyceride hydrolysis by modulating fatty acid chain length and saturation to disrupt lipid structural stability [52]. These results collectively indicate that GL effectively alleviates HFD-induced lipid metabolic disorders by inhibiting lipid synthesis while promoting lipid catabolism and transport. This reprogramming of hepatic metabolism optimizes systemic energy allocation and utilization efficiency, redirecting metabolic flux from lipid storage toward healthier pathways such as protein synthesis and energy oxidation. Ultimately, this shift was reflected in decreased HSI and muscle crude lipid content, alongside increased muscle crude protein content.

Existing research widely recognizes that liver damage induced by HFDs is closely associated with oxidative stress, inflammatory cascade reactions, and the abnormal activation of apoptotic pathways, with the underlying mechanism originating from ROS burst triggered by lipid overload [5,15,53]. On one hand, excessive ROS disrupts the antioxidant system, leading to the accumulation of toxic products such as MDA, which directly attack cellular membrane structures and functional proteins [16]. On the other hand, sustained oxidative stress impairs mitochondrial electron transport chains and endoplasmic reticulum protein folding homeostasis, thereby abnormally activating inflammatory pathways such as NF-κB and NLRP3, as well as apoptotic pathways like CHOP and the Caspase family, collectively exacerbating hepatocyte damage [17,18,54]. Consistent with previous findings [55,56], this study demonstrated that HFDs significantly elevated serum AST and ALT levels, increased hepatic MDA content, and caused severe structural damage to the liver, indicating substantial liver injury induced by HFDs. Meanwhile, gene expression results further corroborated the adverse effects of HFDs. However, GL supplementation effectively reversed the disturbances induced by HFDs, aligning with studies in tilapia [24] and Chinese soft-shelled turtles [25], where GL alleviated hepatic injury through antioxidative and anti-inflammatory mechanisms. Moreover, GL activated the Nrf2/Keap1 signaling axis, enhancing the gene expression of downstream antioxidant enzymes such as *CAT* and increasing T-AOC, thereby enhancing the hepatocyte’s capacity to scavenge ROS at the source. Additionally, the concurrent reduction in hepatic MDA suggests that GL, potentially via its hydrolysis to glucuronic acid, may directly neutralize ROS and lipid peroxidation products through hepatic phase II detoxification reactions, further mitigating oxidative burden [21]. In terms of inflammatory regulation, GL significantly downregulated the expression of pro-inflammatory factors like *IL-1β* while elevating anti-inflammatory factors such as *IL-10* and *TGF-β2*, effectively suppressing the inflammatory cascade by inhibiting the NF-κB signaling pathway. Regarding apoptosis inhibition, similar to reports in chicken hepatocytes [57], GL dose-dependently reduced the expression of *Caspase* while increasing the *Bcl-2/Bax* ratio. Bcl-2 stabilizes mitochondrial membranes and inhibits cytochrome c release, thereby blocking the activation cascade of *Caspase9* and ultimately suppressing the execution of hepatocyte apoptosis [58]. The present results elucidates that GL forms a multi-target intervention network by synergistically enhancing antioxidant defenses, inhibiting inflammatory responses, and blocking mitochondrial apoptotic pathways, thereby systematically alleviating hepatic injury induced by HFDs.

The intestine serves not only as the primary site for nutrient digestion and absorption but also as a critical barrier against pathogen invasion and toxin entry, with its structural integrity being essential for maintaining overall health [59]. Consistent with most existing studies [6,7,9], the results of this study indicate that HFDs significantly compromised the intestinal structural integrity of turbot. In the HFD group, marked disruption of intestinal villi and localized inflammatory cell infiltration were observed. Concurrently, the expression of genes encoding tight junction proteins was significantly downregulated, demonstrating that HFDs successfully induced increased intestinal permeability. Such barrier disruption allows lipid-derived toxic metabolites and endotoxins from the gut to enter the liver via the portal vein, thereby increasing hepatic detoxification burden and triggering systemic low-grade inflammation. This process is considered a key mechanism through which the “gut-liver axis” contributes to or exacerbates liver injury [60,61]. GL is primarily known as a hepatoprotective agent, its protective effects on the intestine have rarely been reported, with only studies in piglets and Chinese soft-shelled turtles demonstrating that GL significantly improves intestinal morphology and enhances barrier integrity [23,25]. Similarly, in this study, GL supplementation effectively reduced serum D-lactate levels and ameliorated intestinal morphology. Moreover, GL up-regulated the expression of tight junction proteins such as Claudin-3 as well as the mucin gene *MUC2*. MUC2 is a major component of the intestinal mucus layer, and its increased expression helps reinforce the chemical barrier, reducing direct contact between harmful substances and the intestinal epithelium [62]. Concurrently, GL significantly suppressed the expression of *MLCK*, a key signaling molecule regulating intestinal permeability that can disrupt intercellular tight junctions by promoting smooth muscle contraction [63]. The downregulation of *MLCK* elucidates, at the signaling pathway level, the mechanism by which GL maintains intestinal barrier tightness. In summary, appropriate doses of GL can effectively enhance intestinal barrier integrity in fish by strengthening physical connections, reinforcing chemical defenses, and inhibiting the MLCK signaling pathway.

The structural stability and functional balance of the intestinal microbiota are fundamental to maintaining gut–liver axis homeostasis, with their compositional changes influencing host liver health through effects on intestinal barrier integrity and modulation of endogenous metabolites [64]. In this study, an HFD did not significantly alter the α-diversity or β-diversity of the gut microbiota in turbot, which differs from some reports indicating that an HFD markedly reduces microbial diversity in fish [9,65]. In contrast, the G600 group supplemented with GL exhibited an increasing trend in α-diversity indices, and its samples showed tighter clustering in β-diversity analysis, suggesting that GL may enhance microbial ecological resilience and structural stability. This observation aligns with previous reports that GL improves gut microbiota diversity in piglets [23]. Although no significant differences were observed in overall diversity, specific shifts were detected at both phylum and genus levels. Consistent with most studies, Proteobacteria, Firmicutes, and Bacteroidetes were the dominant phyla across the three treatment groups, collectively responsible for core functions in nutrient digestion and metabolism [66]. However, at the genus level, an HFD induced a clear trend toward dysbiosis. Among the top 10 genera, an HFD significantly reduced the abundance of *Alcaligenes*, a protease-producing probiotic known to enhance immune activity in fish when used as a feed additive [67], as well as lactic acid bacteria genera such as *Lactiplantibacillus* and *Lacticaseibacillus*, which are recognized for their immunomodulatory and barrier-repairing properties [68]. Concurrently, HFDs significantly increased the abundance of the opportunistic pathogen *Acinetobacter* [69]. GL supplementation partially reversed these microbial alterations. Compared to the NC group, GL significantly suppressed the abnormal proliferation of *Acinetobacter* while increasing the abundance of *Glutamicibacter* and *Akkermansia*. A study in sea cucumbers demonstrated that *Glutamicibacter* improves feed nutritional quality and enhances growth performance and immunity [70]. Moreover, *Akkermansia*, regarded as a next-generation probiotic, is closely associated with the regulation of glucose and lipid metabolism as well as the reinforcement of intestinal barrier function and immunity in fish [71,72].

To further analyze the effects of GL on the gut microbiota, MetagenomeSeq analysis was employed. An HFD significantly increased the abundance of pathogenic bacteria such as *Listeria* [73] and *Austwickia*, while reducing the abundance of short-chain fatty acid (SCFA)-producing bacteria, including *Prevotella* [74], *Turicibacter* [75], *Acidaminococcus*, and *Colidextribacter* [76], as well as beneficial bacteria with immunomodulatory and barrier-repairing potential, such as *Dubosiella* [77] and *Loigolactobacillus* [68]. These shifts collectively indicate gut dysbiosis induced by HFDs, characterized by an increase in harmful bacteria and a decrease in beneficial colonization. Notably, the abundance of *Lachnospiraceae_NK4A136_group*, which associated with glucose and lipid metabolism and barrier function [78], was abnormally elevated in the HFD group; whereas *Prevotellaceae_UCG-001*, positively correlated with barrier damage and inflammation [79], was significantly reduced. These specific changes may be linked to alterations in the intestinal microenvironment caused by HFDs. GL supplementation effectively reversed this imbalance. It significantly inhibited the proliferation of the pathogen *Listeria* while enriching various beneficial bacteria, primarily including SCFA-producing genera (e.g., *Alloprevotella* [80], *Faecalibaculum* [81], *Turicibacter*), bile acid metabolism-related genera (*Ileibacterium* [82]), and bacteria involved in immune and barrier function regulation (e.g., *Allobaculum*, *Alistipes*, *Parabacteroides*, *Rikenella*). Previous studies have confirmed that these genera play key roles in improving metabolism, alleviating inflammation, and repairing the intestinal barrier. For instance, *Allobaculum* promotes butyrate production [83], *Alistipes* ameliorates insulin resistance [84], *Parabacteroides* activates IL-22 to enhance barrier function [85], and *Rikenella* facilitates the absorption of dietary isoflavones [86]. It is noteworthy that a bidirectional interaction may exist between the gut microbiota and the detoxification function of GL. Under high-fat conditions, GL is hydrolyzed into glucuronic acid in the liver, where it conjugates with harmful substances to exert detoxification effects. However, β-glucuronidase (GUS) secreted by the gut microbiota can hydrolyze these glucuronic acid conjugates, potentially releasing toxins and glucuronic acid back into the system, which may affect the therapeutic efficacy of GL [87]. The GL-reshaped microbiota may optimize this process. On one hand, the altered microbial composition could modulate overall GUS enzyme activity; on the other hand, enriched beneficial bacteria (e.g., lactobacilli) may possess the capacity to further metabolize detoxification products, thereby forming a more efficient “synthesis-processing” detoxification loop with the liver [68,88]. However, this hypothesis requires further validation through analysis of intestinal GUS activity and microbial functional profiling. In summary, GL significantly ameliorated HFD-induced gut dysbiosis by reducing harmful bacteria and promoting the colonization of beneficial taxa. This beneficial modulation of the gut microbiota, in synergy with GL’s ability to repair the intestinal physical barrier and mitigate systemic inflammation, collectively constitutes a multidimensional protective network through the gut–liver axis. This network systematically alleviates metabolic injury induced by high-fat diets.

## 5. Conclusions

In summary, GL effectively alleviates HFD-induced growth suppression and immune impairment in turbot, primarily through its multi-target synergistic actions on the liver and intestine. GL systematically reduces lipid deposition and tissue damage caused by HFDs by reprogramming lipid metabolism-related genes, activating the Nrf2/Keap1 antioxidant axis, and suppressing the NF-κB inflammatory and apoptosis pathway. It also effectively restores the intestinal barrier by upregulating the expression of tight junction proteins and *MUC2* while inhibiting the MLCK signaling pathway, and it concurrently remodels the gut microbiota. This dual protective effect on the intestine and liver collectively improved the metabolic microenvironment, thereby alleviating the metabolic damage induced by HFDs. This study provides a theoretical foundation for the application of GL as a functional feed additive and offers a viable nutritional intervention strategy for the prevention and control of nutrition-related metabolic liver diseases.

## Figures and Tables

**Table 1:** Feed composition and nutrient level (% dry weight basis).

Ingredient (%)	Diets				
	PC	NC	G200	G400	G600
Fish meal ^1^	52.00	42.80	42.80	42.80	42.80
Dephenolic cottonseed protein ^2^	10.00	10.00	10.00	10.00	10.00
Wheat gluten meal ^1^	2.00	2.00	2.00	2.00	2.00
Soybean meal ^1^	8.00	8.00	8.00	8.00	8.00
Wheat flour ^1^	19.64	19.64	19.64	19.64	19.64
Fish oil ^1^	5.00	5.00	5.00	5.00	5.00
Soybean oil ^1^	0.00	5.00	5.00	5.00	5.00
Phospholipid oil ^1^	1.00	1.00	1.00	1.00	1.00
Vitamin premix ^3^	1.00	1.00	1.00	1.00	1.00
Mineral premix ^3^	0.50	0.50	0.50	0.50	0.50
Choline chloride	0.30	0.30	0.30	0.30	0.30
Ca(H_2_PO_4_)_2_	0.50	0.50	0.50	0.50	0.50
Y_2_O_3_	0.01	0.01	0.01	0.01	0.01
Calcium propionate	0.05	0.05	0.05	0.05	0.05
Glucuronolactone	0.00	0.00	0.02	0.04	0.06
Microcrystalline cellulose	0.00	4.20	4.18	4.16	4.14
Total	100.00	100.00	100.00	100.00	100.00
Proximate Compositions					
Moisture	3.60	3.56	3.29	4.18	3.98
Crude protein	54.34	47.34	46.99	47.87	45.87
Crude lipid	12.62	17.14	17.27	17.21	17.22
Ash	10.05	8.63	8.74	8.71	8.74
Gross energy (MJ/kg)	20.65	20.79	20.75	20.94	20.47

^1^ From Qingdao Saigelin Marine Biological Feed Co., Ltd. (Qingdao, China). Fish meal: CP 71.59%, CL 11.45%; Wheat gluten meal: CP 86.37%, CL 3.48%; Soybean meal: CP 52.26%, CL 1.31%; Wheat flour: CP 13.70%, CL 3.39%. ^2^ From Xinjiang Jinlan Plant Protein Co., Ltd. (Shihezi, China). Dephenolic cottonseed protein: CP 66.96%, CL 2.83%. ^3^ From Qingdao Master Biotech Co., Ltd. (Qingdao, China). Mineral Premix (per kg): copper, 0.95 g; manganese, 3.03 g; cobalt, 0.1 g; zinc, 16.43 g; iron, 37.14 g; selenium, 0.04 g; iodine, 0.1 g; and moisture 10%. Vitamin Premix (per kg): pyridoxine hydrochloride, 1.10 g; riboflavin, 1.35 g; nicotinamide, 6.75 g; thiamine nitrate, 0.93 g; vitamin D3, 180,000 IU; vitamin A acetate, 1,140,000 IU; menadione, 1.2 g; d-biotin, 47.5 mg; d-calcium pantothenate, 4.5 g; folic acid, 0.465 g; cyanocobalamin, 7.5 mg; dl-α-tocopherol acetate, 7.6 g; inositol, 10 g; l-ascorbic acid-2-phosphate, providing l-ascorbic acid at 16.7 g; and moisture 10%.

**Table 2:** Effects of glucuronolactone on the growth performance.

Indexes	Group				
	PC	NC	G200	G400	G600
IBW (g)	16.7 ± 0.03	16.8 ± 0.09	16.7 ± 0.06	16.7 ± 0.02	16.7 ± 0.04
FBW (g)	47.9 ± 1.15 ^a^	44.2 ± 0.08 ^b^	45.3 ± 0.69 ^b^	45.1 ± 0.66 ^b^	46.8 ± 0.88 ^ab^
SR (%)	100 ± 0.00	99.2 ± 0.83	98.3 ± 0.83	100 ± 0.00	98.3 ± 1.67
WGR (%)	187 ± 6.69 ^a^	163 ± 1.63 ^c^	171 ± 4.62 ^bc^	170 ± 3.92 ^bc^	180 ± 5.11 ^ab^
SGR (%/day)	1.62 ± 0.03 ^a^	1.49 ± 0.01 ^c^	1.53 ± 0.03 ^bc^	1.53 ± 0.02 ^bc^	1.59 ± 0.03 ^ab^
FCR	0.76 ± 0.02	0.79 ± 0.01	0.79 ± 0.01	0.81 ± 0.00	0.77 ± 0.01
FI (%/day)	1.17 ± 0.02 ^ab^	1.12 ± 0.01 ^b^	1.15 ± 0.01 ^ab^	1.19 ± 0.02 ^a^	1.16 ± 0.01 ^ab^
CF	3.64 ± 0.15 ^a^	3.08 ± 0.11 ^b^	3.53 ± 0.13 ^a^	3.52 ± 0.17 ^a^	3.62 ± 0.13 ^a^
HSI	1.15 ± 0.10	1.26 ± 0.08	1.2 ± 0.06	1.09 ± 0.08	1.02 ± 0.09

IBM, initial body weight; FBM, final body weight; SR, survival rate; FCR, feed conversion ratio; FI, daily feed intake; CF, condition factor; HSI, hepatosomatic index. Values are shown as the mean ± SE (*n* = 3). Different superscript lowercase letters within a row denote statistically significant intergroup differences (*p* < 0.05).

**Table 3:** Effects of glucuronolactone on the proximate composition of muscle.

Indexes	Group				
	PC	NC	G200	G400	G600
Moisture (%)	76.4 ± 0.23	76.8 ± 0.18	76.7 ± 0.36	77.1 ± 0.20	77.2 ± 0.18
Crude protein (%)	19.2 ± 0.16 ^a^	18.6 ± 0.08 ^b^	18.8 ± 0.10 ^ab^	18.8 ± 0.11 ^ab^	18.8 ± 0.10 ^ab^
Crude lipid (%)	3.83 ± 0.30	4 ± 0.21	3.88 ± 0.48	3.53 ± 0.22	3.5 ± 0.31
Crude ash (%)	1.32 ± 0.07	1.25 ± 0.01	1.31 ± 0.07	1.25 ± 0.01	1.3 ± 0.03

Values are shown as the mean ± SE (*n* = 3). Different superscript lowercase letters within a row denote statistically significant intergroup differences (*p* < 0.05).

**Table 4:** Effects of glucuronolactone on the serum biochemical parameters.

Indexes	Group				
	PC	NC	G200	G400	G600
LZM (pg/mL)	4.5 ± 0.09 ^ab^	3.87 ± 0.12 ^c^	4.4 ± 0.10 ^b^	4.59 ± 0.07 ^ab^	4.73 ± 0.13 ^a^
ALP (U/L)	9.98 ± 0.94 ^b^	15.1 ± 0.72 ^a^	11.5 ± 0.68 ^b^	10.5 ± 0.44 ^b^	9.5 ± 0.53 ^b^
ACP (U/L)	9.54 ± 0.47 ^c^	10.6 ± 0.28 ^bc^	11.5 ± 0.29 ^ab^	12 ± 0.42 ^a^	10.5 ± 0.42 ^bc^
TC (mmol/L)	1.74 ± 0.09 ^c^	2.6 ± 0.11 ^a^	2.1 ± 0.06 ^b^	1.87 ± 0.08 ^bc^	1.75 ± 0.09 ^c^
TG (mmol/L)	1.21 ± 0.11 ^bc^	1.67 ± 0.09 ^a^	1.41 ± 0.04 ^b^	1.12 ± 0.03 ^c^	1.08 ± 0.06 ^c^
GLU (mmol/L)	0.46 ± 0.06 ^b^	0.75 ± 0.08 ^a^	0.5 ± 0.08 ^b^	0.5 ± 0.08 ^b^	0.4 ± 0.05 ^b^
AST (U/L)	6.38 ± 0.46 ^a^	5.65 ± 0.27 ^a^	3.09 ± 0.40 ^b^	3.22 ± 0.30 ^b^	3.19 ± 0.18 ^b^
ALT (U/L)	3.57 ± 0.59 ^b^	6.27 ± 0.72 ^a^	4.51 ± 0.59 ^ab^	4.07 ± 0.86 ^b^	3.55 ± 0.31 ^b^
D-LA (nmol/L)	30.5 ± 1.18 ^b^	39 ± 1.60 ^a^	35.7 ± 1.34 ^ab^	31.9 ± 1.70 ^b^	31.1 ± 2.26 ^b^

LZM, Lysozyme; ALP, alkaline phosphatase; ACP, acid phosphatase; TC, total cholesterol; TG, triglycerides; GLU, glucose; AST, aspartate aminotransferase; ALT, alanine aminotransferase; D-LA, D-lactic acid. Values are shown as the mean ± SE (*n* = 3). Different superscript lowercase letters within a row denote statistically significant intergroup differences (*p* < 0.05).

## Data Availability

The dataset is available on request from the authors. The 16S rRNA gene sequence data have been submitted to the NCBI SRA depository within BioProject PRJNA1428554 (https://dataview.ncbi.nlm.nih.gov/object/PRJNA1428554?reviewer=aktq1h1ntl85obmd8s2h0as858, accessed on 6 April 2026).

## Supplementary Materials

The following supporting information can be downloaded at: https://www.mdpi.com/article/10.3390/antiox15040472/s1. Table S1: Sequence of the primers used for real-time qPCR; Figure S1: Rarefaction curve (A) and species accumulation (B) of the 16S sequencing data.
