# Supplementary material for: Glucuronolactone Alleviates Metabolic Stress Induced by High-Fat Diet in Turbot (Scophthalmus maximus L.)
Source: Antioxidants (Basel). 2026 Apr 10;15(4):472. doi: 10.3390/antiox15040472 (PMC13113150; doi:10.3390/antiox15040472)
Supplement: Supplementary file 1 [file antioxidants-15-00472-s001.zip › antioxidants-4192083-supplementary.pdf]

Glucuronolactone alleviates metabolic stress induced by high-fat diet in turbot (*Scophthalmus maximus L.*)

Ping Wang [1, †], Luyao Zheng [1, †], Liping Zhu [2], Kecai Chen [2], Dongsheng He [2], Jingjing Zhao [1], Houguo Xu [3], Kangsen Mai [1], Yanjiao Zhang [1] *

[1] *Key Laboratory of Aquaculture Nutrition and Feed, Ministry of Agriculture and Rural Affairs, The Key Laboratory of Mariculture, Ministry of Education, Ocean University of China, Qingdao 266003, China*

[2] *Shandong Engineering Research Center for Natural Product Metabolic Engineering and Synthetic Biology, Weifang 255178, China*

[3] *Yellow Sea Fisheries Research Institute, Chinese Academy of Fishery Sciences, 106 Nanjing Road, Qingdao 266071, China*

[†] Ping Wang and Luyao Zheng contributed equally to this work.

*Corresponding author:
Email: yanjiaozhang@ouc.edu.cn (Yanjiao Zhang)

Table S1 Sequence of the primers used for real-time qPCR.

Gene	Forward primers (F)	Reverse primers (R)	Accession number
β-actin	CGTGCGTGACATCAAG GAG	AGGAAGGAAGGCTGG AAGAG	AY008305.1
TNF-α2	GGACAGGGCTGGTACA ACAC	TTCAATTAGTGCCACG ACAAAGAG	XM_035629860 .2
IL-1β	CGCTTCCCCAACTGGT ACAT	ACCTTCCACTTTGGGT CGTC	AJ295836.2
IL-10	TTTCGAAAGTCCGTTT GCGT	CATCGGACTTGAGCTC GTCG	XM_035632547 .2
INF-γ	ATGTCTGTGACCTTGGT GCC	TGTTGCCCAGACATTC CTGT	DQ400686. 1
NF-κB	ACACTGCTGAGCTGAA GATC	CTCTGAGCCCATCAGG GTC	MF370855
IL-8	GTCTGAGAAGCCTGGG AGTG	TCTTCGCAGTGAGAGT TGCC	HQ872499.1
TGF-β2	TCGCAGCACAAAAACC ATCG	CAGCCCAGGTCCTTTT GGA	XM_035608274
Caspase3	TTCTGCCATTGTCTCTG TGC	GCCCTGCAACATAAAG CAAC	KY979513.1
Caspase7	TCTGCAATGTCCTCAAC GAG	TTGCGACCATGTAGTTG ACC	XM_035612079
Caspase9	CCCAGGACATGATCGA CGAG	ACAATGGGAAGGCTCG ACTG	XM_035644988 .2
Bcl-2	TTCCTCAACTCTCAAA GCACAATTC	ATTACACTCGCTCGCCA TTCC	MN782168
Bax	AGCATCTTTGCTGACG GGAT	GCGCTCTCTGATGACC TGAA	MN782169
CAT	TATCTTCGTCCGCACTG TTG	AGAAACCCAGCCTCAC TTTG	MG253621.1
SOD	CATCATCAGCACGTCTC TCC	GGCTCCATAGTCGTAG GTCA	MG253620.1
GPX	CCCTGATGACTGACCC AAAG	GCACAAGGCTGAGGA GTTTC	AWP02885.1
Keap 1	ACAGAGTGCTTGACAG AATGC	GGCTCTCTAGCGTGTA GCTG	XP_035492645. 2
HO-1	GAGTCACGTCAGGGCA GAAA	GGGGAAGTAAATGGGT GCGA	JX453446. 1
Nrf2	ACAAGCCTTCCTTCAC ACCC	CTGGCACTTCAGCCGT TAGA	MT023796. 1
SREBP1	GCCATTGACTACATCCG TTAC	CATCAGCCTGTCCATCT ACTTC	MH174964.1
PPAR-γ	AAGTGACGGAGTTCGC CAAGA	GTTCATCAGAGGTGCC ATCA	XM_035631101 .2

ACC	GTTCCAACCAAGGCTCCGTATGAC	TCTGCCGACCACCACACTCTG	XM_035625110.2
SCD	TCCACAGCCGGAGAACATTT	CGGTTTGGGCGATGCAAAA	XM_035612833.2
DGAT2	ATTTACACGGCCTGGCTCAT	GAGGACCTTCTTCCACCTTGTT	XM_035625396.2
FAS	ATCCACAGAGCCACCATCCTACC	CCAGAATGCTCACCTTACCACTCAC	XM_035612874.2
PPAR-α1	CCCTGATAACACCTTCCTCTTTCCC	TGTCTCGGTCGTCTTGATGTCCTG	XM_047336020.1
ACOX1	TCCGCTACAGTGTCGTTC	AGTCTCCCTGGCTGATGT	XM_035638509.2
CPT1	GCCTTTCAGTTCACCATCACA	ATGCGGCTGACTCGTTTCTT	XM_035614266.2
HSL	TGGCACGAATTAGGCAGGTT	GCATTGGCCTTTTGCGAAGA	XM_035619927.2
LPL	CAGCAGGATCACAGGTATGGAT	GGACAAGGTGCTCTGGTCAT	XM_035648263.2
CYP7A1	ACTGGGAGGTGGATGGTATCTTCG	GCCTGCCTCGCTTGACACTTATC	XM_035635553.2
ApoB100	CCATCCCTGCACCAATGAGT	TGCCACCAGAGTGTTTGTCAT	XM_035617338.2
CD36	CCTGGACGTGGAACCCATAA	GCCGTCTCATTCAGCCAAAC	XM_035648654.2
MTP	TGGAAACCTGCCCCATGAAA	TCACGTCAGCAGATTGAGCC	XM_035637540.2
Claudin-3	GCCATCTTCGCGTGCGTT	TCCGGAACTCCCGTATCCTC	KU238182. 1
Claudin-7	AAGTACGAGTTTGGGTCGC	TCCTTGCTACTTGGGGGTCT	MF370858. 1
ZO-1	CGCCACCAGCAAAACCAGTC	CGATGAAGATGCCCACG TCG	KU238185. 1
Occludin	CGTCGCCATCTTCGCGTG	CGGAGCCATATCCCATTCCC	KU238182. 1
MLCK	CACTGCCCAGTGTAAGAGCA	GCCTCCTCTTCTCGCAGTTT	XM_035614007.2
MUC2	GTTTCAAACCACCTGCGACC	GCATGGGAACACTGACGAGA	KU238186. 1

TNF-α2, Tumor necrosis factor-alpha 2; *IL*, Interleukin; *INF-γ*, Interferon-gamma; *NF-κB*, Nuclear factor kappa-B; *TGF-β2*, Transforming growth factor-beta 2; *Caspase*, Cysteinyl aspartate specific proteinase; *Bax*, BCL-2-associated X protein; *Bcl-2*, B-cell lymphoma-2; *CAT*, Catalase; *SOD*, Superoxide dismutase; *GPX*, Glutathione peroxidase; *Keap 1*, Kelch-like ECH-associated protein 1; *HO-1*, Heme oxygenase-1; *Nrf2*, Nuclear factor erythroid 2-related factor 2; *SREBP1*, Sterol regulatory element-binding protein 1; *PPAR-γ*, Peroxisome

proliferator-activated receptor gamma; *ACC*, Acetyl-CoA carboxylase; *SCD*, Stearoyl-CoA desaturase; *DGAT2*, Diacylglycerol O-acyltransferase 2; *FAS*, Fatty acid synthase; *PPAR-α1*, Peroxisome proliferator-activated receptor alpha 1; *ACOX1*, Acyl-CoA oxidase 1; *CPT1*, Carnitine palmitoyltransferase 1; *HSL*, Hormone-sensitive lipase; *LPL*, Lipoprotein lipase; *CYP7A1*, Cholesterol 7-alpha hydroxylase; *ApoB100*, Apolipoprotein B100; *CD36*, Cluster of differentiation 36; *MTP*, Microsomal triglyceride transfer protein; *ZO-1*, Zonula occludens-1; *MLCK*, Myosin light-chain kinase; *MUC2*, Mucin 2.

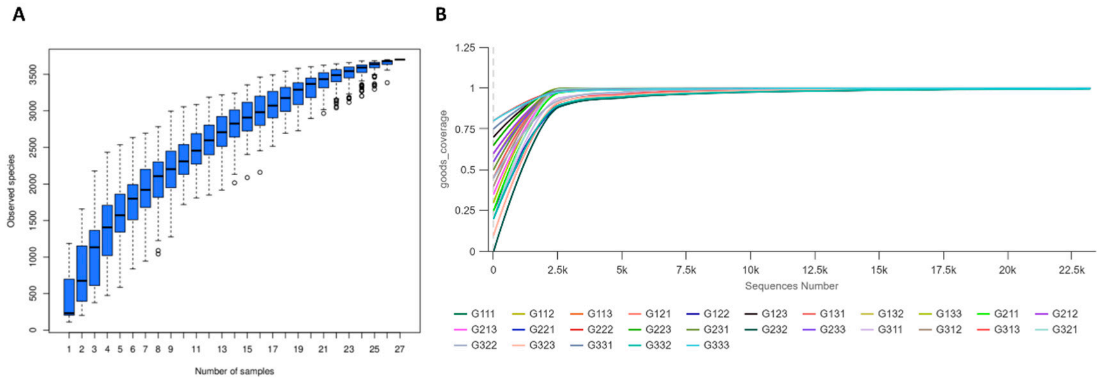

Figure S1 Rarefaction curve (A) and species accumulation (B) of the 16S sequencing data.
